# Cardiovascular magnetic resonance in an adult human population: serial observations from the multi-ethnic study of atherosclerosis

**DOI:** 10.1186/s12968-017-0367-1

**Published:** 2017-07-18

**Authors:** Kihei Yoneyama, Bharath A. Venkatesh, David A. Bluemke, Robyn L. McClelland, João A.C. Lima

**Affiliations:** 1 Department of Cardiology, Johns Hopkins University, Baltimore, MD USA; 2 Radiology and Imaging Sciences, National Institutes of Health Clinical Center, Bethesda, MD USA; 3 Department of Biostatistics, University of Washington, Seattle, WA USA; 4 St. Marianna University School of Medicine, Kawasaki, Japan; 5 Professor of Medicine, Radiology and Epidemiology, Johns Hopkins Hospital, Johns Hopkins University, Blalock 524D1, 600 North Wolfe Street, Baltimore, MD 21287 USA

**Keywords:** Ageing, Fibrosis, Heart failure, Cardiovascular disease, Torsion

## Abstract

The Multi-Ethnic Study of Atherosclerosis (MESA) is the first large-scale multi-ethnic population study in the U.S. to use advanced cardiovascular magnetic resonance (CMR) imaging. MESA participants were free of cardiovascular disease at baseline between 2000 and 2002, and were followed up between 2009 and 2011 with repeated CMR examinations as part of MESA. CMR allows the clinician to visualize and accurately quantify volume and dimensions of all four cardiac chambers; measure systolic and diastolic ventricular function; assess myocardial fibrosis; assess vessel lumen size, vessel wall morphology, and vessel stiffness. CMR has a number of advantages over other imaging modalities such as echocardiography, computed tomography, and invasive angiography, and has been proposed as a diagnostic strategy for high-risk populations. MESA has been extensively evaluating CMR imaging biomarkers, as markers of subclinical disease, in the last 15 years for low-risk populations. On a more practical level, some of the imaging biomarkers developed and studied are translatable to at-risk populations. In this review, we discuss the progression of subclinical cardiovascular disease and the mechanisms responsible for the transition to symptomatic clinical outcomes based on our findings from MESA.

## Background

The Multi-Ethnic Study of Atherosclerosis (MESA) is a prospective study sponsored by the National Heart, Lung, and Blood Institute (NHLBI) of the National Institutes of Health. The major objective of MESA was to evaluate the mechanisms that underlie the development and progression of subclinical cardiovascular disease (CVD) among asymptomatic individuals in the general population. MESA was primarily designed to study the progression of subclinical CVD and the mechanisms responsible for the transition to symptomatic clinical outcomes [1]. In brief, between July 2000 and August 2002, 6814 men and women, 45 to 84 years of age—who identified themselves as Caucasian, African American, Hispanic, or Chinese, and who were free of clinically apparent CVD were recruited. Cardiovascular magnetic resonance imaging (CMR) was performed on 5004 participants as part of the baseline examination and 3015 underwent a follow-up CMR between 2010 and 2012 (year-10).

CMR is a noninvasive medical test that creates detailed pictures of the beating heart and vessels to look at their structure and function. In MESA, CMR was used to accurately quantify volume and dimensions of all four cardiac chambers, systolic and diastolic ventricular and atrial function, assess extent of replacement and interstitial fibrosis, and measure aortic structure and function, to evaluate patterns of cardiac structural remodeling and the prevalence of myocardial fibrosis across the life course, identify the myocardial mechanical consequences of remodeling, explore ventricular-arterial coupling and the role of arterial stiffness in CVD, and to study the prognostic importance of CMR biomarkers.

In this review, we discuss the progression of subclinical CVD and the mechanisms responsible for the transition to symptomatic clinical outcomes based on our findings from MESA.

### Left ventricular (LV) remodeling across the life course

The observed variations in the structure of the left ventricle might be attributable to differences in demographic variables (e.g., age, gender, and race), traditional risk factors (e.g., LV overload by blood pressure or atherosclerosis), other medical conditions, socioeconomic status, and genetic influences in general populations. In MESA, LV wall thickness by CMR was associated with gender, race, body size, and LV function [2]. Chinese participants had lower volumes and mass than other ethnic groups among normal participants without known CVD risk factors [3]. LV volumes and mass were significantly greater for men than women, and, more spherical shape of LV was seen in women as compared to men [4–6].

**Fig. 1:**
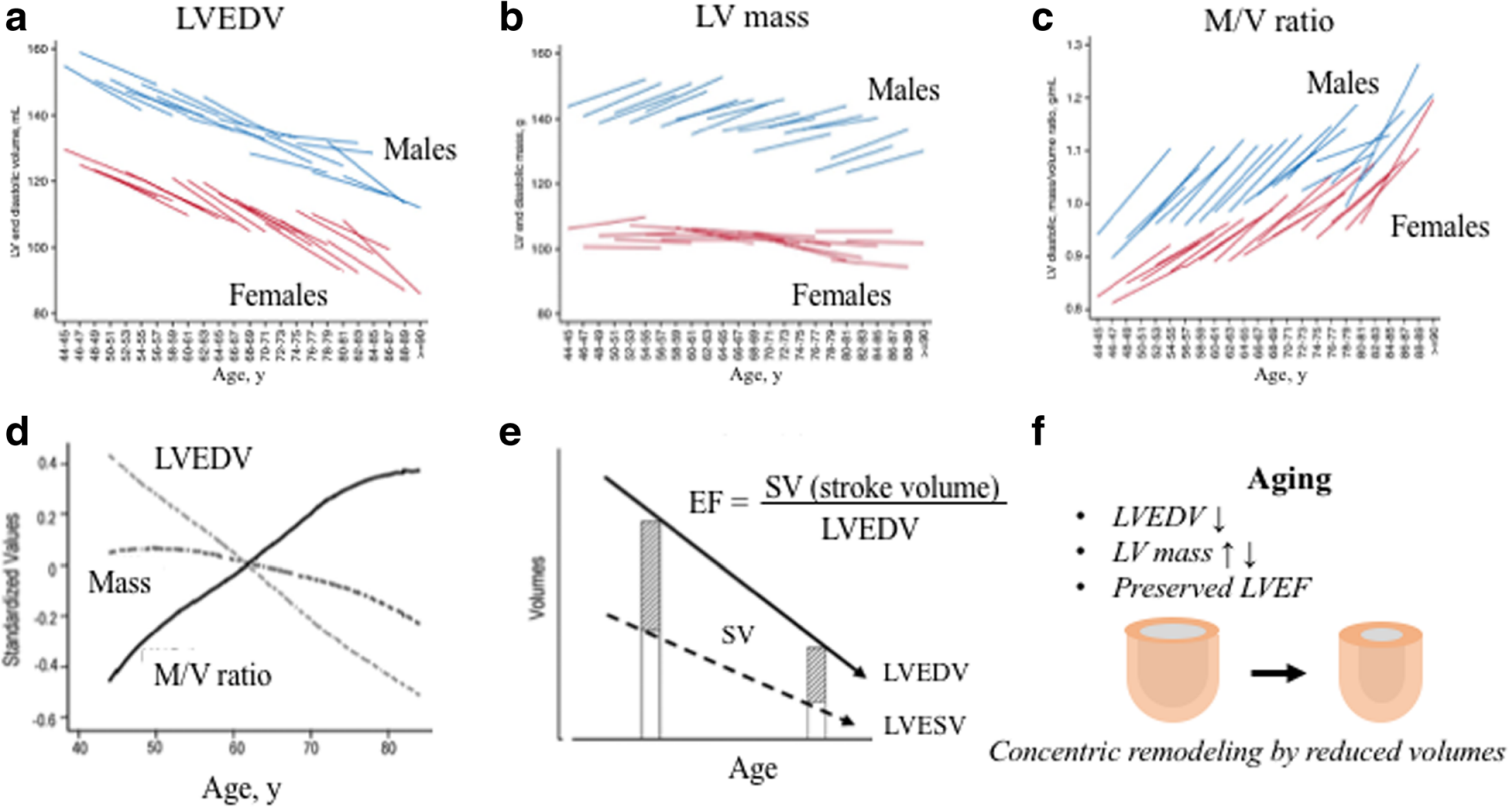
The natural history of myocardial function in an adult human population. LVEDV statistically significantly decreased over 10 years for each age category **a** and LV mass increased in Men **b** and M/V ratio increased **c** despite LV mass did not progressively increase **b** and **d**. Although SV progressively falls, LVEF maintains due to progressive decline in LV volumes **e**. Aging is associated with the development of a concentric remodeling pattern secondary to a progressive decline in LV volume **f**. LV = left ventricular; LVEF = left ventricular ejection fraction; LVEDV = Left ventricular end diastolic volume; LVESV = Left ventricular end systolic volume; M/V = mass-to-volume ratio Figures prepared based on data from Eng et al. [8], and Cheng et al. [5]

The average age of populations around the globe is increasing; as a result, an increasing number of older individuals become afflicted by CVD [7]. While age is one of the most powerful risk factors for CVD, it is important to understand the LV adaptation mechanisms that function against the altered cardiovascular system during aging in human adult life. The findings of MESA CMR related to aging are summarized in Fig. 1. The MESA serial longitudinal study confirmed that a pattern of LV remodeling across the life course is concentric remodeling with progressively reduced LV volumes resulting in an increased LV mass-to-volume ratio. Specifically, we see a significant age-related longitudinal increase in LV mass in men and a slight longitudinal decrease in women over 9 years follow-up; additionally, longitudinal decreases in LV end-diastolic volume and stroke volume were seen in both men and women, leading to significant increases in adverse LV concentric remodeling (increased mass-to-volume ratio) in both men and women [8]. The LV ejection fraction (LVEF), however, was maintained with age due to a progressive decline in LV end-diastolic volume (Fig. 1) [5].

Aging prolongs exposure to CVD risk factors, therefore, understanding the influences of adverse LV remodeling by risk factors in the general population is important. In cross-sectional studies, CVD risk factors were associated with LV concentric remodeling (i.e., increased blood pressure, body mass index, body fat, insulin resistance, and presence of hypertension and diabetes), even though variants of LV remodeling patterns were observed with each CVD risk factor (i.e, hypertensive participants had concentric remodeling with greater LV volumes, whereas diabetic participants had concentric remodeling with lower volumes) [4, 6, 9–12]. In addition, a higher ankle-brachial index, traffic exposure, and genetic variation were associated with higher LV mass [13–15]. Higher physical activity was associated with higher LV mass but not with concentric remodeling, and higher activity was associated with lower heart rate [16]. MESA longitudinal studies confirmed some of the findings of the cross-sectional studies by showing that changes in CVD risk factors are associated with changes in LV remodeling, e.g., weight loss, reduced blood pressure, and reduced heart rate were associated with a smaller increase in age-related concentric remodeling [11, 12].

### LV remodeling and cardiac events

**Fig. 2:**
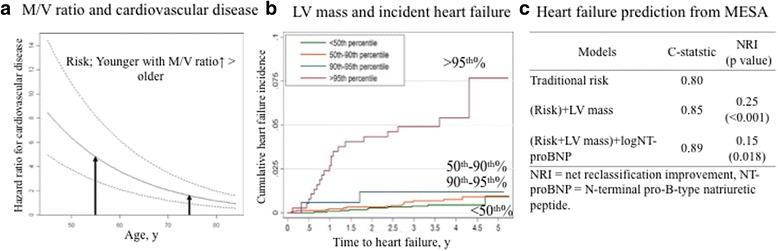
Left ventricular remodeling and incident cardiac events in MESA. The risk of incident cardiovascular disease is greater for those individuals who develop the concentric remodeling at a younger compared with older age (*black arrows*, **a**). Higher LV mass (hypertrophy) is associated with incident heart failure **b**. Additional LV mass and log NT-pro BNP produces the largest increased in c-statistic and improves the NRI beyond traditional risk factors **c**. Figures prepared based on data from Cheng et al. [5], Bluemke et al. [21], and Chahal et al. [22]. Abbreviations as in Fig. 1

Progressive LV remodeling may identify individuals at high risk for cardiac events in the general population. For example, in contrast to the natural history of concentric remodeling, the risk of incident CVD is greater for those individuals who develop concentric remodeling at a younger age (Fig. 2a). This pattern of ventricular remodeling appears to confer significant CVD risk, especially when present earlier in life [5].

Heart failure (HF) is an important contributor to the burden and cost of national healthcare expenditures, and with the aging of worldwide populations, the estimated prevalence and cost of care for HF will increase markedly for all sex and racial/ethnic subgroups [7, 17]. The pattern of hypertrophy is related to pressure overload leading to concentric hypertrophy and volume overload leading to eccentric hypertrophy.

The Framingham study has suggested that early identification of LV hypertrophy by electrocardiogram (ECG) or echocardiography may provide an opportunity to prevent the development of cardiac events, however, the MESA CMR study found that ECG had low sensitivity (10% to 26%) for detecting CMR-defined hypertrophy [18], and that CMR is highly accurate using 3-dimensional ventricular size and shape compared to conventional echocardiography [19]. The predictive value of CMR for heart failure (HF) events exceeded that of coronary artery calcium (CAC) and carotid intima-medial thickness (IMT) for both men and women [20].

In MESA, higher LV mass at baseline (≥95th percentile compared to the reference group of the <50% percentile) is strongly associated with incident HF with a hazard ratio of 8.6 (95% confidence interval: 3.9 to 19.9) (Fig. 2b) [21]. The addition of N-terminal pro b-type natriuretic peptide (NT-pro BNP) with LV mass produced the largest increase in the c-statistic over the basic traditional risk assessment for predicting HF (Fig. 2c) [22]. Extreme LV sphericity was associated with higher incidence of HF and atrial fibrillation [23]. The strengths of MESA include high-quality assessments of multiple risk factors for HF including traditional risk factors and biomarkers, which are responsible for the high predictive value of the MESA HF score [22].

### LV replacement fibrosis in the general population

Late gadolinium-enhancement imaging (LGE) can be used to detect replacement or focal myocardial fibrosis (scar) with a high degree of accuracy [24, 25]. CMR LGE represents end-organ damage and worse cardiac prognosis among patients with cardiomyopathy [26, 27]; identification of LGE might significantly improve risk stratification in the general population.

From a community-based cohort in Iceland with a median age of 76 years, the prevalence of unrecognized myocardial infarction (MI) detected by CMR with LGE was 17% (157/936), and was associated with increased mortality similar to recognized MI, compared to individuals with no MI [28]. In MESA, of 1840 participants with a mean age of 68 years, 7.9% had myocardial scars defined by focal LGE, of which 78% (114/146) were undetected by ECG or by clinical adjudication [29]. The following individual risk factors were associated with higher odds of myocardial scar: age, male sex, body mass index, current smoking, and hypertension at baseline in 2000 to 2002 [29]. Importantly, CAC score by CT at baseline was associated with a typical ischemic scar pattern in 2009 to 2011 [29]. These suggest that unrecognized MI appears to represent a phenotype of coronary heart disease related to atherosclerosis risk factors.

From a clinical longitudinal study of patients with idiopathic, dilated cardiomyopathy (non-ischemic) receiving optimal medical therapy, LV systolic function decreased during a 24-month follow-up in patients with myocardial scar at baseline, while reverse remodeling was observed in patients without myocardial scar [30]. MESA reported results similar to that in a high-risk population, namely that asymptomatic individuals with prevalent LGE had an enlarged left ventricle (concentric hypertrophy in women and LV dilatation in men) with progressively declining LVEF; conversely, individuals without myocardial scar maintained LV function [31]. Therefore, the presence of replacement fibrosis was associated with changing LV volumes and reduced function over time even in individuals in lower-risk populations, and who may be at risk to develop asymptomatic HF regardless of whether fibrosis was ischemic or atypical.

### LV interstitial fibrosis in a general population

Although LGE CMR allows for the assessment of replacement myocardial fibrosis, it is limited in the evaluation of diffuse interstitial fibrosis. T1 myocardial mapping—a fairly new technique that can be used to identify the exact T1 value of tissue—enables direct myocardial signal quantification, characterization of myocardial tissue composition and assessment of interstitial fibrosis, and correlates with interstitial fibrosis as assessed by means of invasive biopsy [24, 25].

Our study, using contrast T1 found the following two main results: First, the age-related remodeling pattern of left ventricule—which is a progressive decline in LV volumes leading to concentric remodeling—is seen with progressive LV diffuse fibrosis. In other words, persions with either greater LV mass or without concentric remodeling are likely to have less interstitial fibrosis on average [31, 32]. Cardiac aging, characterized by shrinking LV volume with diffuse interstitial fibrosis, can support the pathological finding of an age-related increase in myocyte deaths accompanied with reductions in coronary vasculature through the cardiac life course [31, 33]. Second, hypertension contributed to interstitial fibrosis [31, 32]. This also supports the pathological findings of increases in the extracellular matrix by accumulation of perivascular fibrosis and fibrosis of the endomysium and perimysium in hypertensive heart disease [34].

**Fig. 3:**
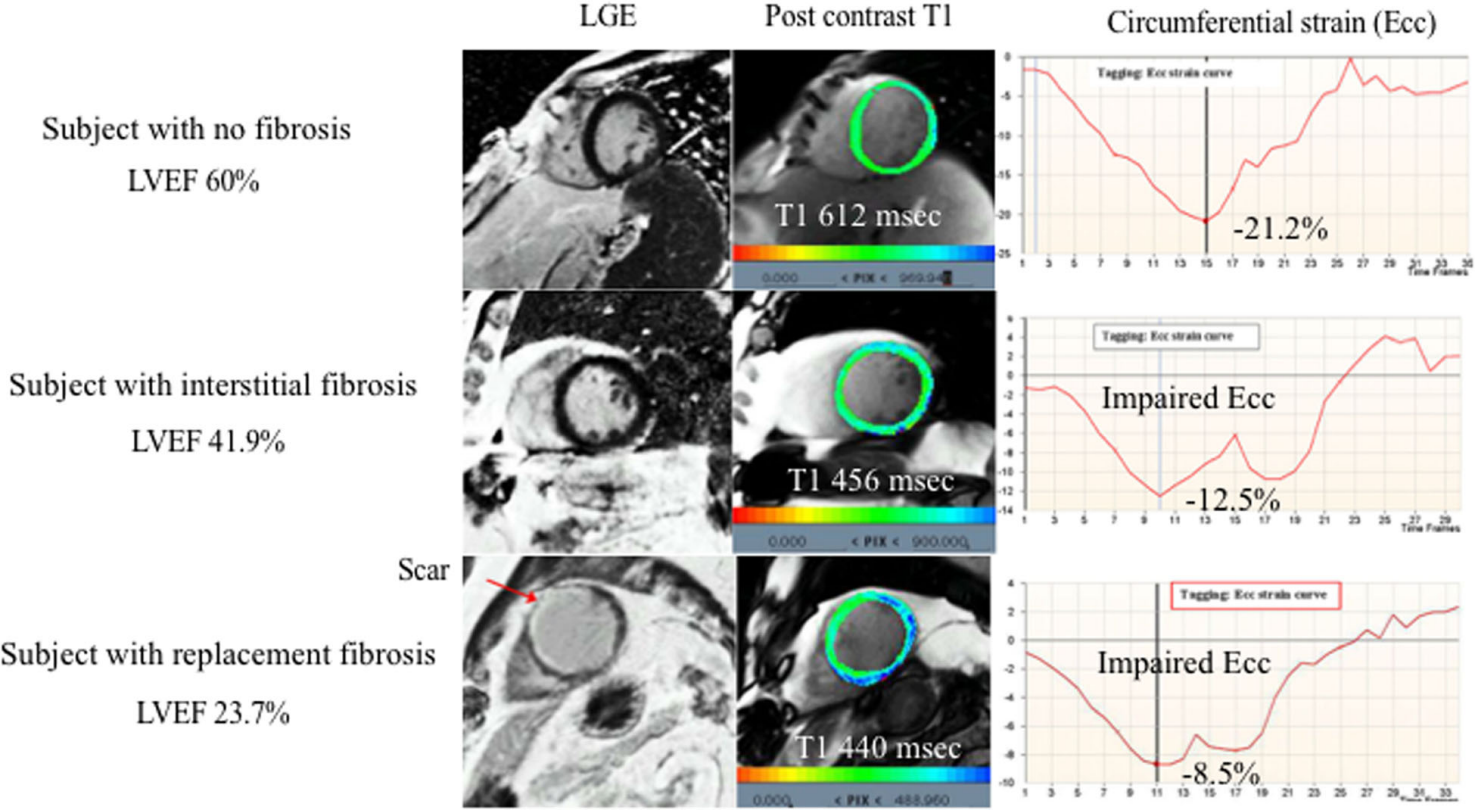
Fibrosis and left ventricular function in MESA. The subject without a LGE-defined scar had a relatively lower post contrast T1 time at 25 min with altered regional myocardial shortening and LVEF compared to the subject without an LGE-defined myocardial scar and no evidence of extra-cellular expansion. The subject with a LGE-defined replacement fibrosis had a progressively reduced systolic function. Figures prepared based on data from Donekal et al. [35]. LGE = late gadolinium-enhanced image; LVEF = left ventricular ejection fraction

In MESA, both replacement and interstitial fibrosis are important determinants of LV function, as shown in Fig. 3. Interestingly, men had a linear decline in LV systolic function with interstitial fibrosis, whereas women had a progressive decline in LV diastolic (not systolic) function with fibrosis [31, 35]. Perhaps restrictive filling with eventual diastolic dysfunction—due to progressive diffuse myocardial fibrosis with fewer myocytes (related to aging)—could progress to a stiffer left ventricule. In addition, men had progressively increased interstitial fibrosis with burden of CVD risk factors, while women were likely to have more interstitial fibrosis than men independent of CVD risk factors [32, 36]. Of note, these results concur with a previous epidemiology study finding that diastolic HF is more common in elderly men and women than in younger patients [37].

### Cardiac deformation imaging using tagged CMR

CMR tissue tagging provides precise quantification of incipient myocardial dysfunction through the assessment of myocardial strain and torsion, and has been used as the reference standard to validate two-dimensional speckle tracking echocardiography.

**Fig. 4:**
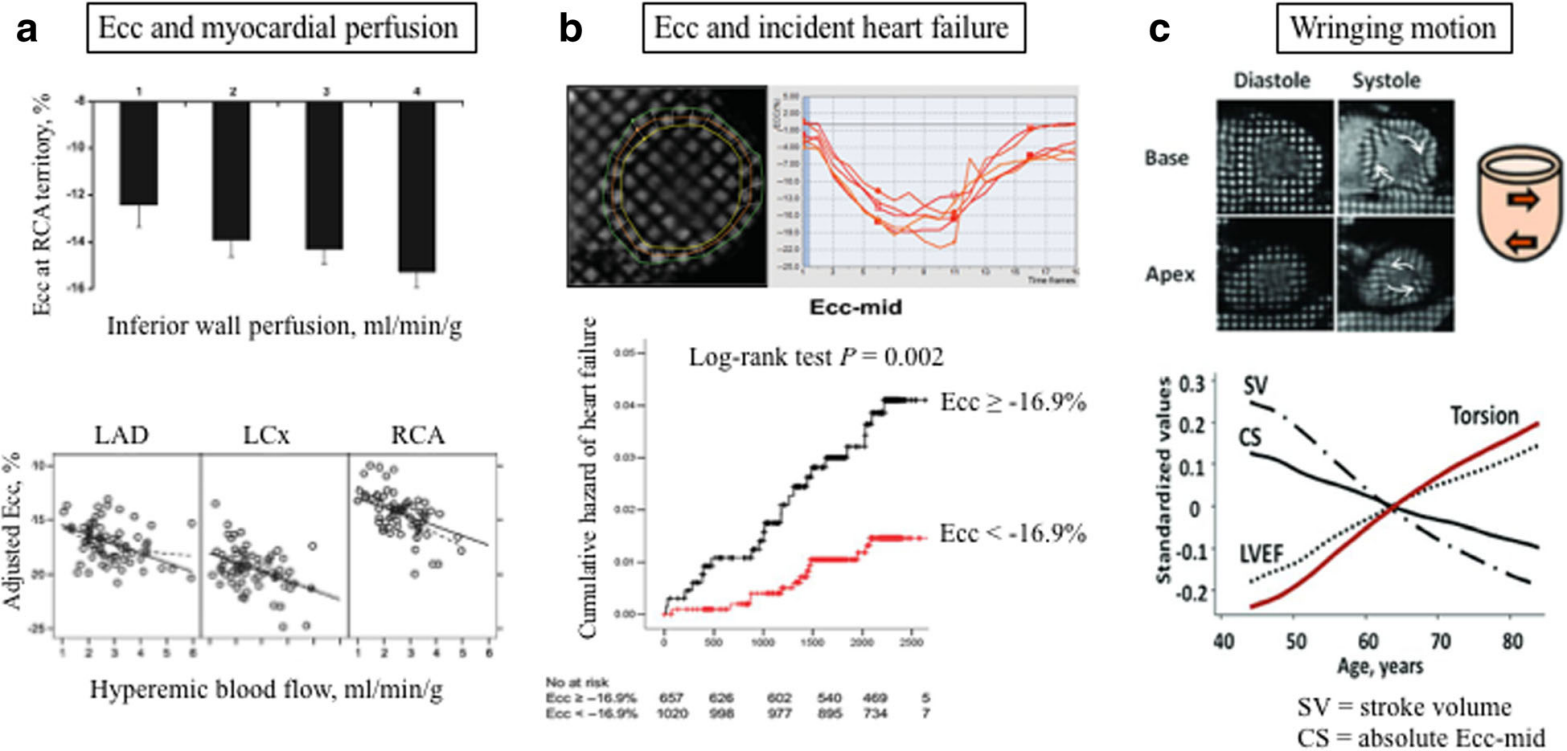
Cardiac deformation imaging using tagged CMR in asymptomatic individuals. Impaired Ecc is associated with lower myocardial perfusion and blood flow **a** Impaired LV circumferential shortening (Ecc) provides predictive value for incident heart failure during 5.5 years follow-up **b** LV torsion, however, is greater with older age despite lower stroke volume and myocardial shortening **c**. LV wringing motion might represents a compensatory mechanism for systolic dysfunction to maintain LVEF. Figures prepared based on date from Rosen et al. [38], Choi et al. [45], and Yoneyama et al. [6]. LAD = left anterior descending artery; LCx = left circumflex artery; RCA = right coronary artery. Abbreviations as in Fig. 1

In the MESA population, reduced regional peak systolic circumferential strain was associated with impaired regional myocardial perfusion in participants who did not have relevant coronary artery stenosis (Fig. 4a) [38]. This suggests that impaired myocardial shortening enhances the ability to identify early subendocardial ischemia and has a potential role in risk stratification beyond traditional LVEF. For example, worse circumferential myocardial strain has been observed to be associated with LV hypertrophy, concentric remodeling, subclinical atherosclerosis, older age, male sex, hypertension, resting higher heart rate, smoking, and metabolic syndrome [5, 6, 39–44], and has been shown to have incremental predictive value for HF events beyond the traditional LVEF (Fig. 4b) [45]. Diastolic function from circumferential strain curves showed a powerful independent ability for the prediction of HF and atrial fibrillation over an 8-year follow-up period [46]. In addition, tagged CMR provides further insight into the depth of the cardiac pump mechanism in humans. For example, it has been noted that perhaps the discrepancy between the age-associated increase in LVEF and the decline in circumferential shortening can be explained by the enhanced wringing motion of the left ventricule assessed by tagged CMR (Fig. 4c) [6], which represents a human compensatory mechanism to maintain LVEF in age-related concentric remodeling.

**Fig. 5:**
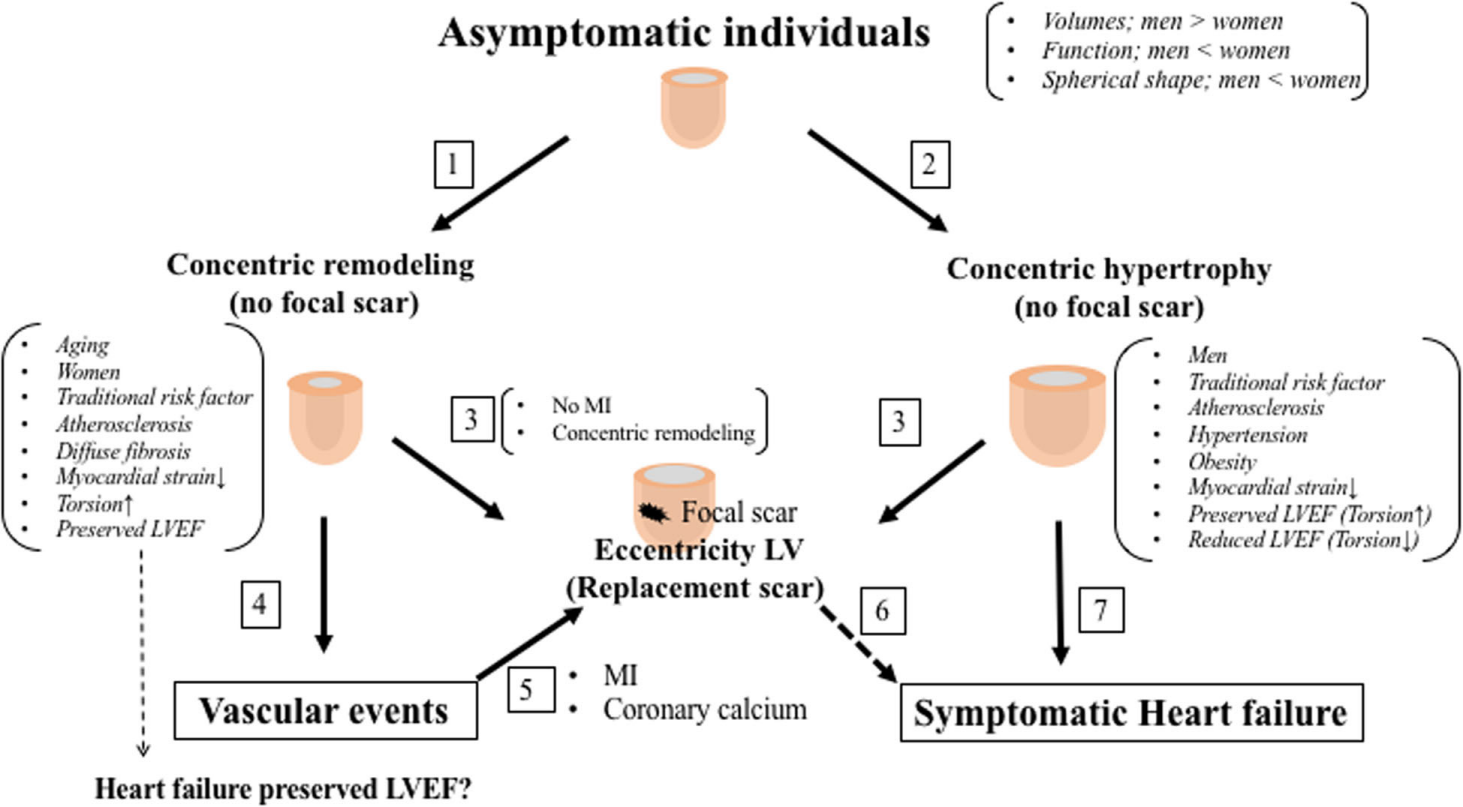
Potential pathways in the progression from asymptomatic to symptomatic cardiovascular events from MESA CMR remodeling and fibrosis data. Aging leads to concentric remodeling (higher M/V ratio and maintained LV mass) without no focal scar (pathway 1) [5, 6, 69]. Typically hypertension progresses to concentric hypertrophy (higher M/V ratio and LV mass) without focal scar (pathway 2) [6, 31]. The pathway from concentric remodeling and hypertrophy to replacement fibrosis without MI (pathway 3) [31]. Individuals with concentric remodeling can develop vascular events with preserved LVEF (pathway 4) [5, 21]. Individuals with concentric remodeling with MI or severe coronary calcification can contribute to replacement fibrosis (pathway 5) [29]. Individuals with concentric hypertrophy can develop symptomatic vascular events either with replacement scar (pathway 6) [28] or without (pathway 7) [21, 22]. Black arrows depict MESA CMR results, and thicker dot arrow, a known pathway. MI = myocardial infarction. Abbreviations as in Fig. 1

Finally, progression of symptomatic events from asymptomatic individuals is summarized in Fig. 5.

### A novel CMR marker for LV trabeculation

Clinically, LV noncompaction is a myocardial disorder characterized by excessive LV trabeculae. Although a high degree of noncompacted (trabeculated) myocardium in relationship to compact myocardium (trabeculated to compact myocardium [T/M] ratio > 2.3) has been associated with a diagnosis of LV noncompaction, MESA found that 43% of subjects without cardiac disease or hypertension had a T/M ratio > 2.3 in at least 1 myocardial segment [47]. Using fractal dimension, which is an automated assessment for trabecular quantification that is relatively insensitive to compacted myocardium wall thickness, the LV was more trabeculated in African-American and Hispanic participants than white participants, and smoothest in Chinese-American participants [48]. MESA also confirmed in a longitudinal observation study that a greater extent of LV trabeculation was not associated with an absolute decline in LVEF during the approximately 10 years of the MESA study. Our findings suggest that in subjects with a low pre-test probability for cardiomyopathy and marked trabeculation, greater trabeculation is not related to ejection fraction in asymptomatic subjects [49]. However, the relationship of high degrees of trabeculations to regional function and other remodeling mechanisms has not yet been determined [50].

### Right ventricle in a general population

CMR is the preferred method for evaluating right ventricular structure and function due to its crescent-shape in cross-sectional images. In MESA, older age was associated with lower RV (right ventricular) mass and higher RV ejection fraction; men had greater RV mass and larger RV volumes than women, but had lower RV ejection fraction; African-Americans had lower RV mass than whites, whereas Hispanics had higher RV mass [51]. Traffic exposure was associated with higher RV mass, i.e., participants who lived in the same neighborhood for several years had the strongest associations between nitrogen dioxide and RV mass [52]. Participants with RV hypertrophy had a significant increase in the risk of HF or death that was independent of LV mass, and had a stronger association with the outcome in the setting of lower LV mass [53].

### Left atrial (LA) measurements by tissue-tracking CMR in asymptomatic individuals

In normal sinus rhythm, LA performance is characterized by a complex of three basic functions: reservoir function (collection of pulmonary venous flow during LV systole), conduit function (passage of blood to the LV during early diastole), and active booster pump function (augments LV filling during LV late diastole) [54, 55]. Hoit’s review of clinical studies examining LA size and function found that LA ejection fraction, which is volume assessment, is a promising measure for predicting CVD in a patient population [56].

Tissue-tracking on LA wall from cine CMR can provide an alternative and additional assessment of risk stratification or diastolic function beyond LA volume assessment. Data from a clinical study using this technique on 169 patients with a history of atrial fibrillation indicated that lower LA strain and strain rate during LV diastole (impaired LA reservoir function) is significantly associated with a prior history of stroke, and that the incremental value of LA strain for diagnosis of stroke is further increased by adding LA global strain to LA volume [55]. Similarly, in the MESA population, asymptomatic individuals who developed HF had a lower LA strain during LV diastole (impaired LA reservoir function) and a higher minimum LA volume (impaired pump function and volume) than those without HF events. The deteriorations in LA performance appeared to robustly predict HF events independent of LV mass and NT-pro BNP [57].

**Fig. 6:**
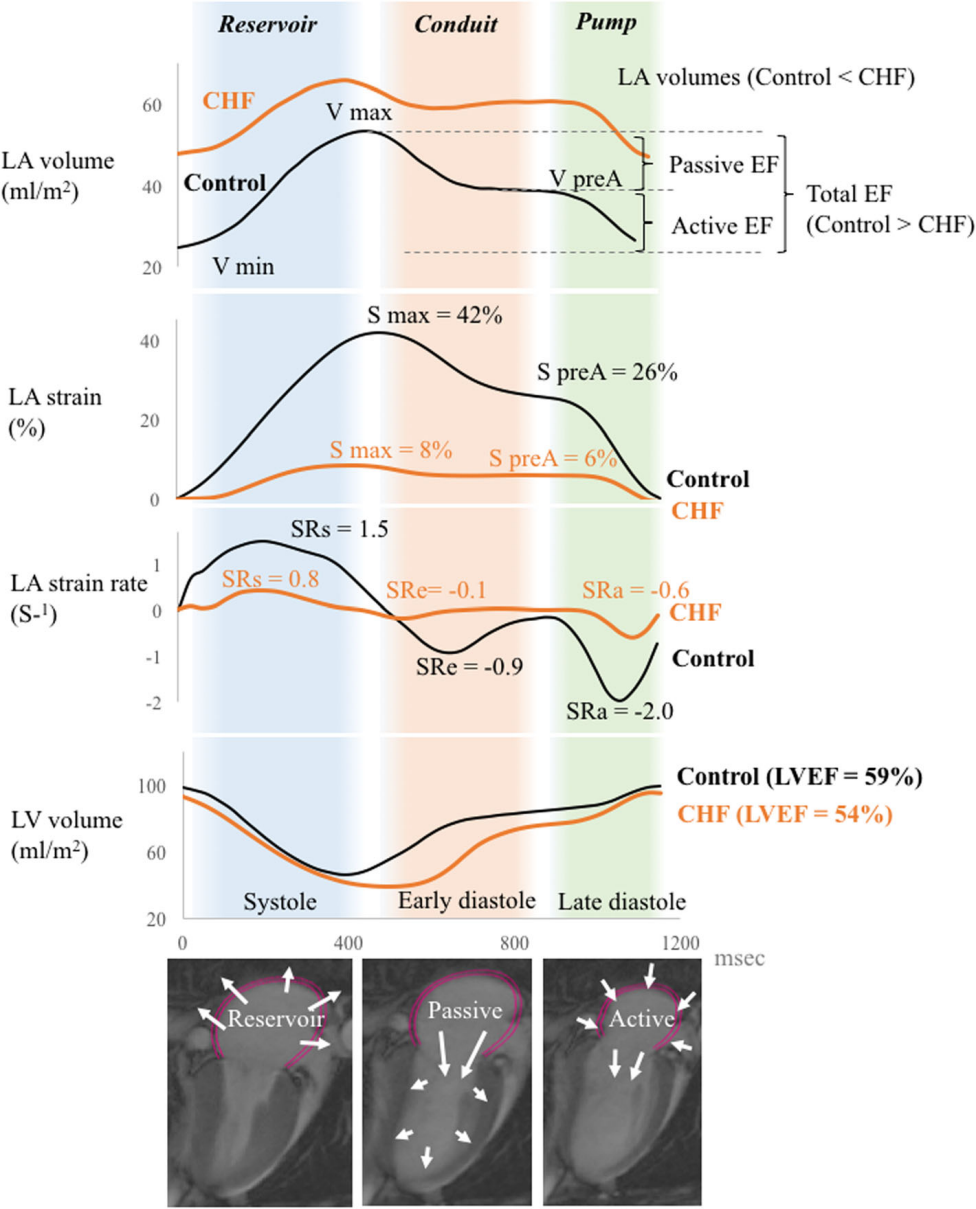
Left atrial measurements by tissue-tracking CMR in asymptomatic individuals. LA volume, LA strain, LA strain rate, and LV volume in a asymptomatic participant free of cardiovascular daises as a control (*block line*) and a MESA participant who developed CHF (*orange line*). A case of CHF had a relatively greater LA volumes with impaired LA strains (S max and S preA) and LA strain rates (SRs, SRe, and SRa) than a control. Volumes are index to body surface area. LA function was analyzed by using a tissue-tracking method with semiauto mated software (multimodality tissue tracking [MTT] version 5.0; Toshiba, Tochigi, Japan). CHF; congestive heart failure; LA = left atrial; LV = left ventricular; S max = maximum strain; S preA = pre atrial contraction strain; SRa = strain rate at atrial contraction; SRe = strain rate at LV early diastole; SRs = maximum strain rate, V max = maximum indexed volume; V min = minimum indexed volume; V pre A = pre-atrial contraction indexed volume

Our study also reported the following: first, a presence of replacement scar on LV was associated with all three basic LA performance functions including an impaired reservoir function (lower maximum LA strain and strain rate), a conduit function (lower absolute strain rate), and a pump function (lower absolute strain rate), but was not significantly associated with total LAEF. Second, LV diffuse myocardial fibrosis assessed by contrast T1 was associated with an impaired reservoir function (lower LA strain) and a pump function (lower LA strain rate) but was not significantly associated with LA volumes [58]. These findings suggests that tissue-tracking on the LA wall can be a more sensitive marker to detect early myocardial disease among asymptomatic individuals despite the fact that LAEF might decrease, as documented in advanced decompensated cardiac disease (Fig. 6).

### Vascular imaging using CMR in MESA

CMR has a distinct advantage over ultrasound in that three-dimensional visualization of the vessel wall and identification of components on the atherosclerotic plaque are possible. CMR has the unique ability to assess aortic geometry and function, representing vascular stiffness by vessel distensibility and pulse wave velocity with high reproducibility [59, 60].

**Fig. 7:**
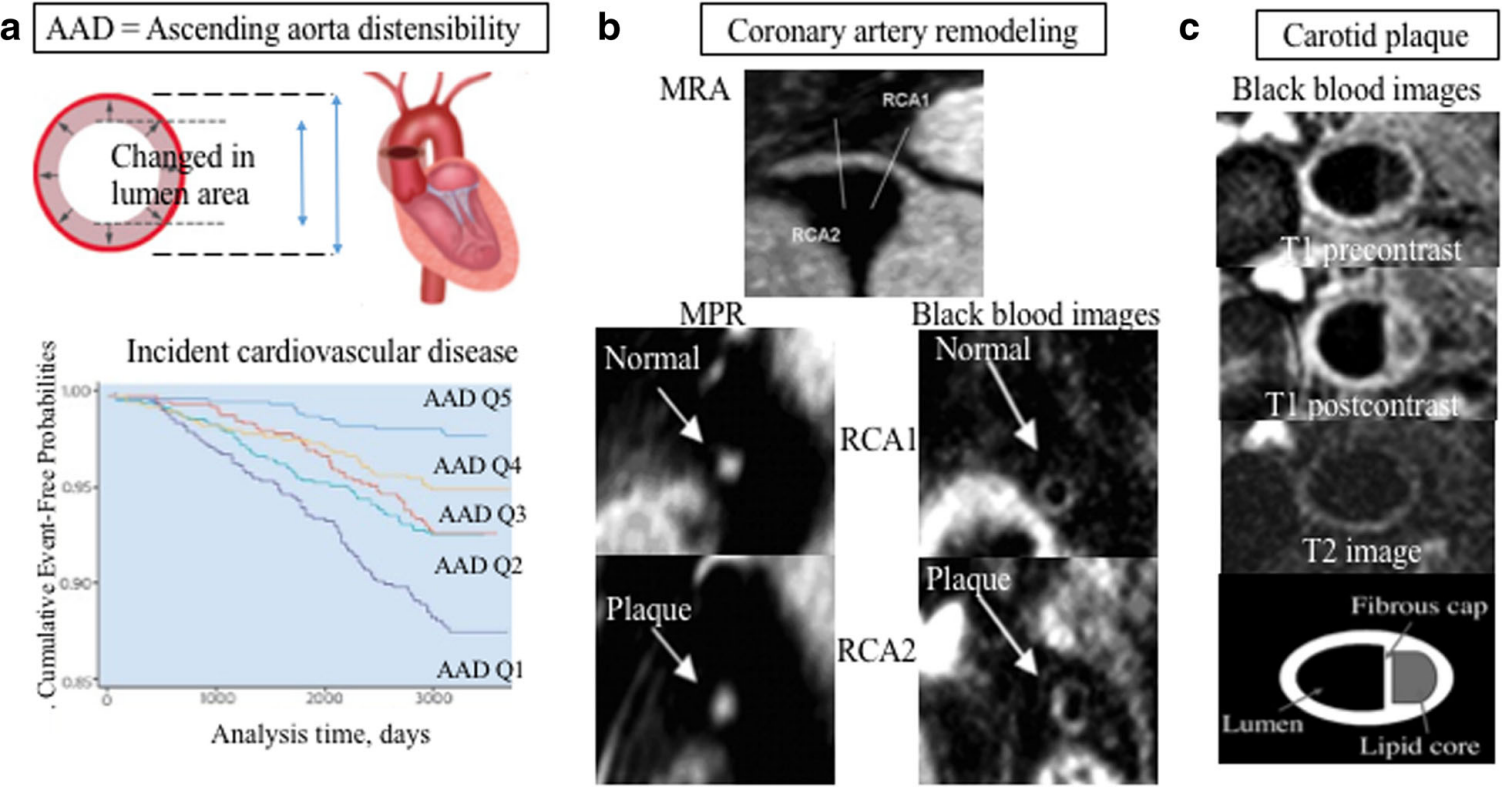
Aortic imaging using CMR in in asymptomatic individuals. Decreased proximal aorta distensibility predicted incident cardiovascular disease **a** MRA without contrast shows no significant stenosis in the proximal RCA, and black blood images shows normal wall and eccentric plaque **b** Black blood images obtained the carotid artery, and identify lipid core **c**. Figures prepared based on date from Redheuil et al. [62], Miao et al. [66] and Wasserman et al. [67]

Of over 3500 MESA participants with a mean age of 61 years, ascending aorta diameter was increased with age; higher aortic stiffness as measured by aortic distensibility was associated with hypertension, larger left ventricle, and incident CVD events (Fig. 7a) [60–63]. Individuals with high aortic stiffness had an almost 4-fold increase in risk of incident CVD compared to individuals with preserved aortic distensibility if they had a low-to-intermediate-risk CVD profile [62].

In a longitudinal MESA study over 10 years of follow-up, aortic stiffness was associated with wall thickness of the aorta, which increases by 0.032 mm/year [63]. Age, smoking, and increased blood pressure were associated with increases in aortic stiffness, but reduction in blood pressure was associated with less increase in the stiffness [64]. The loss of proximal aortic function was a robust marker of vascular alteration, particularly in individuals with a lower traditional CVD risk profile as compared to those with a higher risk profile.

Atherosclerotic coronary artery plaque rupture is a key event leading to acute coronary syndromes. Black-blood coronary arterial wall CMR provides a unique new method to nonivasively image and assess the morphological features of the coronary arteries (Fig. 7b). MESA has reported the ability of CMR to identify coronary artery remodeling as an early marker of subclinical coronary atherosclerosis among participants with zero CAC score by computed tomography. The spatial resolution of CMR, however, needs improvement [65, 66].

MESA also has used black-blood sequences to noninvasively characterize carotid-artery atheroma composition (Fig. 7c). Using this technique, a lipid core was seen in 71% as selected from this asymptomatic population who had thickened carotid walls (>1.5 mm by CMR), and plasma total cholesterol rather than other CVD risk factors, was strongly associated with lipid core presence by CMR [67]. In addition, carotid artery remodeling and lipid core presence were independent predictors of incident CVD events in MESA [68]. Consequently, vascular imaging features by CMR might be important predictors of cardiovascular risk in asymptomatic individuals.

## Conclusion

MESA is one of the first large-scale population studies to use advanced CMR imaging techniques among asymptomatic individuals, and to follow up with repeated CMR examinations as part of a large multi-ethnic population study. A dominant pattern of LV remodeling across the life course is concentric remodeling by progressively reduced LV volumes. Progressively increased LV concentric remodeling is associated with interstitial fibrosis and is a marker of CVD events. Abnormal remodeling with LV hypertrophy is associated with replacement fibrosis, and a high risk for developing HF. Replacement scar, however, in more than half of the participants with scar by CMR LGE is undetected by ECG or by clinical adjudication.

Assessing LA and vascular geometry and function can be alternative tools to detect early stage subclinical disease. These CMR markers may significantly improve risk stratification in the general population. Finally, further advances in imaging are required to understand the role of risk factors and the progression of symptomatic disease pathways in the asymptomatic.

## Abbreviations

CAC: Coronary artery calcium
CMR: Cardiovascular magnetic resonance
CVD: Cardiovascular diseases
HF: Heart failure
LA: Left atrial
LGE: Late gadolinium enhancement
LV: Left ventricular
MESA: Multi-Ethnic Study of Atherosclerosis
MI: myocardial infarction
RV: Right ventricular
